# The Sense of Dignity at the End of Life: Reflections on Lifetime Values through the Family Photo Album

**DOI:** 10.3390/bs10110177

**Published:** 2020-11-23

**Authors:** Ines Testoni, Vera Baroni, Erika Iacona, Adriano Zamperini, Shoshi Keisari, Lucia Ronconi, Luigi Grassi

**Affiliations:** 1 Department of Philosophy, Sociology, Pedagogy and Applied Psychology (FISPPA), University of Padova, 35131 Padova, Italy; baroni.vera@alice.it (V.B.); erika.iacona@unipd.it (E.I.); adriano.zamperini@unipd.it (A.Z.); 2 The Faculty of Social Welfare and Health Sciences, University of Haifa, Haifa 5560249, Israel; skeisari@gmail.com; 3 Statistical Services, Psychology Multifunctional Center, University of Padova, 35131 Padova, Italy; l.ronconi@unipd.it; 4 Institute of Psychiatry, Department of Biomedical and Specialty Surgical Sciences, University of Ferrara, 44121 Ferrara, Italy; luigi.grassi@unife.it

**Keywords:** dignity therapy, human values, PhotoTherapy, end of life, palliative care

## Abstract

This study focuses on the dimensions of dignity, linking Chochinov’s Dignity Therapy to Schwartz’s Theory of Values. The use of family photo albums has enriched the application of dignity therapy. Seven terminal patients in home-based palliative care participated in the therapeutic intervention. To measure the effects of the intervention, we administered the Edmonton Symptom Assessment Scale and the Patient Dignity Inventory, then, at the end of the meetings, collected the opinions of participants, available nurses, and relatives who attended the sessions. The resulting generativity documents were then analyzed through thematic analysis, which revealed three main themes linked to both fundamental values and the dimensions of dignity: The relationship between continuity of self and myriad values in the context of family relationships; personal dignity as characterized by the values of personal success, hope, and wisdom; and hope and generativity. The fourth theme reflected the participants’ final judgements on the intervention, which were highly positive and greatly encouraged further use of photographs in similar therapeutic interventions. The assessment protocol highlighted a significant decrease in tiredness amongst the participants and a trend towards a significant decrease in drowsiness after the intervention.

## 1. Introduction

Numerous studies have shown that the underlying reason for patients’ desire for euthanasia is a depreciation in life value due to the loss of dignity and hope [1,2]. On the basis of these results, Harvey Max Chochinov formulated his Model of Dignity in the Terminally ill Patient (MDTP) [3,4,5], which describes three main factors related to patients’ perception of lost dignity: Illness-related concerns, which pertain to the consequences of one’s physical and psychological condition; dignity-conserving repertoires, which relate to the psychological and spiritual dimensions of one’s life; and the social dignity inventory, which reflects the social and relational dimensions of one’s life. On the basis of this theoretical framework, Chochinov et al., developed an innovative, brief, and individualized psychotherapy called Dignity Therapy (DT) [6], which aims to reduce psychosocial–existential suffering, increase quality of life, and support a sense of meaning, purpose, and dignity [7] amongst people approaching the end of life by offering them a final opportunity to address important issues or to talk about things they would like to remember as death approaches [8,9].

DT consists of a semi-structured audio-recorded interview, which is transcribed and edited, to produce the final “generativity document”. Once produced, the generativity document is returned to patients who will bequeath it to friends or family members. The process of construction of this document facilitates the remembrance of images of their own life and to dwell on the most significant experiences. It is based on MDTP [10] and involves three elements [11]: The first is the preparation of a concrete generativity document—effectively the transcript of the audio-recorded interview—which reflects the patient’s personal sense of dignity/identity; the second is the tenor of care, which is based on Client-Centered Therapy (CTT) [12,13]; the third is the 11-question, semi-structured interview, which addresses the preservation of dignity and is divided into eight sub-themes (continuity of self; role preservation; generativity/legacy; maintenance of pride; hopefulness; autonomy/control; acceptance; resilience/fighting spirit).

Studies with cancer patients undergoing palliative care have shown that DT enhances the value of life at the end of life by strengthening one’s sense of dignity and improving one’s relationships with loved ones, who also benefit from the intervention by learning how to better manage their mourning [6,10,14,15,16,17]. Because DT is adaptable to different cultures and contexts, there are many ongoing DT studies and clinical application programs occurring in different countries. It has even been used with different modalities and vulnerable populations [15,18,19,20].

The most important part of DT—the creation of a generativity document—may be supported by the use of real photos [21]. In fact, considering the patients’ possible difficulties, the use of such a medium may facilitate recollection and better structuring of the narrative, as reported in other PhotoTherapy studies [22,23]. Photo-based-therapy focuses on the particular emotional-communicative qualities of photographs to allow people to express their concerns and difficulties [24,25]. The use of photos along with the interview is also known as “Photo elicitation” [26]. Using visual stimuli within interviewing prompts emotional connections to memories and provides more meaningful accounts during the interview [27]. From an interpretative point of view, although the format of the DT interview is standardized, photographs allow for infinite dialogical narratives, enabling psychotherapists to identify the most salient aspects of emerging issues [28,29].

A recent study on generativity documents highlighted the importance of individual values. In particular, it showed how the values that patients considered to be the most important during their lives served as fundamental pivots in their DT narratives [30]. Based on Schwartz’s intercultural model of values [31,32], which describes the principles guiding people’s behavior as desirable and trans-situational, this study considers dignity as a function of those values that participants cherished over the course of their lives. In fact, one’s sense of dignity may be construed as a fundamental value that influences all dimensions of the person. Starting from the definitions of value given by Rokeack [33] and Kluckhohn [34], Schwartz and Bilsky [35] defined values as the basis on which people’s individualistic and/or collectivistic interests develop, thereby guiding their attitudes and behaviors. The Quasi-Circumplex Model of Human Values (QCMHV) [31,36,37] describes an integrated system of ten basic values that respond to three types of universal needs (very similar to the three dimensions of dignity described by DT): Biological needs, requisites of coordinated social interaction, and demands of group survival and functioning. Each of the values occupies a precise position based on their relation to four superordinate value categories, which form two bipolar/reciprocal opposition dimensions: (1) Openness to change, which contains self-direction and stimulation, vs. conservation, which involves conformity, tradition, and security; and (2) self-enhancement, which includes power and achievement, vs. self-transcendence, which comprises universalism and benevolence. Hedonism, understood as personal gratification of the senses, is another value category that has no opposing dimensions [31,38]. QCMHV is considered universal because the values it describes are present in almost all cultures, assuming the same meaning and order [35,39].

Assuming that the use of a photo album could aid the recollection of facts and emotions and facilitate reflection on the values that shaped the narrated experiences, the principal aims of this study were to (1) confirm whether photo-mediation was suitable for DT with end-of-life cancer patients, (2) identify the role that values played in the narratives within the generativity documents, and (3) recognize the role of the values described in Schwartz’s model through qualitative analysis of the generativity documents.

## 2. Materials and Methods

### 2.1. Participants

There were seven participants in this study, all of whom were interviewed at home, where they could easily access the family photo album. The criteria for inclusion were: Experiencing a life-threatening condition; life expectancy not exceeding six months; awareness and understanding of the prognosis; over 18 years of age; full cognitive functioning; common language between the participant and the interviewer (excellent ability to understand and speak Italian language by the participant and the interviewer). Possible candidates were identified by the hospice health care staff, specifically palliative physicians gave the interviewer information about the prognosis and cognitive status after informed consent of the patient. Once the candidates had been selected, the prospect of DT was formally presented to them by the interviewer adequately prepared by therapists previously trained in that method. All participants (and their family members) reviewed and signed the informed consent document.

The sample’ characteristics are shown in Table 1, in which eighty percent of the participants were male. The average age was 63 years (range 44–79, *SD* = 15); the average schooling was 11 years; 70% were Italian, 30% Albanian or Moldovan. The 2 foreign participants spoke the Italian language perfectly since they had been living in Italy for more than 20 years and were perfectly integrated. Seventy percent were married with children; 70% were retired. Six people were suffering from cancer, and one from Amyotrophic Lateral Sclerosis. The first person we interviewed was a 44-year-old Albanian man with 12 years of schooling and a married worker with two children; four meetings were held with him, and his generativity document was intended for his sister. The second person we interviewed was a 44-year-old Moldovan woman with 12 years of schooling and a married worker with two children; five meetings were held with her, and her generativity document was intended for all her family members. The third participant was a 69-year-old Italian man with 5 years of schooling and a married pensioner with two children; eight meetings were held with him, and his generativity document was intended for his family members. The fourth participant was a 77-year-old Italian man with 10 years of schooling and a married retiree with three children; six meetings were held with him, and his generativity document was intended for his wife and children. The fifth person was a 66-year-old Italian man with 13 years of schooling and a married retiree with no children; six meetings were held with him, and his generativity document was intended for his wife, brother, and a family friend. The sixth person was a 79-year-old Italian man with 11 years of schooling and a married retiree with two children; six meetings were held with him, and his generativity document was intended for himself. The seventh participant was a 79-year-old Italian woman with 16 years of schooling and a single retiree with one child; nine meetings were held with her, and her generativity document was intended for her son and sister.

### 2.2. Methodology

The study utilized a mixed methods design with repeated measures (before and after DT). The following scales were administered as part of the quantitative assessment (on average, 6 days between pre- and post-test).

The Edmonton Symptom Assessment Scale (ESAS) [40]—which was validated in Italy by Moro et al. [41]—considers the presence and severity/intensity of ten symptoms (physical, psychological, and one at the patient’s discretion), evaluated on a 0–10 scale. Because of its simplicity and agility, the instrument can be administered repeatedly to capture the evolution of symptoms over time, or it can be used for short-term assessments.

The Patient Dignity Inventory (PDI)—which was developed from the MDTP [10] and later validated in Italy by Ripamonti et al. [42]—evaluates distress according to the terminal patient’s perception of dignity. The instrument is composed of 25 items that are scored on a 5-point Likert scale, and it investigates three primary domains: Illness-related concerns, including level of independence and symptoms of distress; dignity conserving repertoire, including dignity-conserving perspectives and practices; and perceived social dignity.

At the end of the treatment, a further question was added regarding participants’ opinion on DT. Participants in this case included patients and those who supported patients in their treatment (relatives and caregivers).

The pre-test and post-test scores of these assessments were analyzed by Wilcoxon’s test, a non-parametric test which allowed us to examine the ranks of the difference form pre-test to post-test. This choice was determined by the small sample size.

On average, the at-home pre- and post-tests were performed over 23 days (range 11–28).

The qualitative assessment was administered through the DT semi-structured interview [6], during which participants were asked to mentally retrace the photo album of their life focusing on the most significant moments. Once the interview was over, patients were given the opportunity to enrich the generativity document with images from the family photo albums that supported the narration. Participants then showed the most important photos to the interviewer and eventually chose their preferred photos for inclusion in the generativity document. All the contents of the interviews were transcribed verbatim, resubmitted to participants for corrections and, during the penultimate meeting, returned for final approval. During the last meeting, the final version of the generativity document was handed to the patient, who was asked to indicate the final recipient. The number of DT sessions per patient varied from 4 to 9, since they were adapted to the psychophysical conditions of the patient.

The corpora were analyzed using thematic analysis, which allows researchers to identify relevant issues arising in the dialogue. The structure of the DT questionnaire presented a series of sequential topics, though these were sufficiently flexible to allow sources to be examined in terms of their principal content [40,41,43]. The interpretative process involved six main phases: Preparatory organization; reading and re-reading to recognize key concepts; coding data; interpreting themes; searching for alternative explanations; and producing the final report [42,44,45]. The process was realized by identifying categories that only became clear as the analysis proceeded (‘bottom-up’), exploring connections between QCMHV, explicit statements, and implicit meanings [46,47]. The analysis was performed with ATLAS.ti, a computer program that facilitates the identification of thematic networks [48].

## 3. Results

The Shapiro–Wilk normality test indicates non-significant results (*p* > 0.05) for all measures except for Nausea, Discomfort, and Other Symptom of ESAS, but the normality test can have insufficient power to produce useful results with a small sample sizes as in our study, and consequently, we adopted a no parametric test (Wilcoxon’s test) to evaluate changes over time. The results of Wilcoxon’s test are shown in Table 2. For the items of ESAS, we can observe a decrease after the intervention, positive ranks were always higher than negative ranks except for the items Appetite and Anxiety, but their decrease was not statistically significant (all *p* > 0.05). For two items, the decrease was close to be significant, Fatigue and Drowsiness (*p* = 0.066). Both items showed only positive ranks, and 4 subjects decreased their score form pre-test to post-test, and the other 3 subjects showed no change in their score over time. For all domains and for the total score of PDI, no significant changes were observed, positive and negative ranks were always very similar, indicating no specific trend, to increase or to decrease, over time.

In terms of the qualitative assessment, each narrative produced an average of 9800 words, suggesting that the photographs helped the participants to answer the interviewer’s questions. A total of 155 photographs were used in this process, and all of them portrayed the participants together with their closest friends and relatives. There were no differences in terms of fundamental values between native Italian and non-native speakers’ participants.

Table 3 shows the relationships between the categories in Chochinov’s MDTP and Schwartz’s QCMHV.

### 3.1. The First Theme: The Relationship between Continuity of Self and Myriad Values in the Context of Family Relationships

The most well-represented dignity dimension was the continuity of self, which affected almost all the value categories of the QCMHV. This dimension was particularly characterized by the following values: Stimulation (meaning ‘living an exciting life,’ or ‘full of strong emotions’; frequency [*f* = 13]); self-direction (meaning ‘achieving personal growth/maturity’ or ‘being independent’; *f* = 7 and 5, respectively); and benevolence (interpreted as ‘the tendency to establish stable and mature emotional relationships’ [*f* = 7], ‘being helpful to others’ [*f* = 6], or ‘the tendency to establish strong, sincere, supportive friendships’ [*f* = 4]).

Our analysis revealed that almost all the participants’ most cherished values related to their families. With respect to stimulation, two aspects consistently appeared to be related: Emotional intensity and having lived a life full of challenges (*f* = 8), as exemplified by the second person interviewed’s narrative: “It was a very difficult but also exciting life, especially when I found out I was pregnant! This changed my life and made it wonderful! In fact, I had wanted to be a mother for so many years, and I had resigned myself to being sterile. Then, everything changed and nothing was the same. Everything was difficult but also fantastic!” Benevolence was mostly understood as the tendency to establish stable and mature sentimental relationships (*f* = 7), as the seventh participant’s account highlighted: “My family has been a source of intense and positive affection for me, capable of lasting [a long] time. My son represents everything; he is my greatest treasure and I am happy that he is capable of reciprocating this affection.” Benevolence was also associated with ‘security’ and ‘cohesion’, as demonstrated in the fourth participant’s narrative: “Our family has been the source of all the good things in my life. My wife helped me with everything. Without her, I could have done nothing…and the unity of the family came, first of all, from our unity as a couple.” The benevolence of one’s family relationships was also linked to security in terms of deteriorated health (*f* = 4), as exemplified by the third participant’s account:

“My wife enables me to endure my days. When I wake up in the morning, I don’t think about illness. When the pain starts to tear me up, everything becomes more difficult, but she supports me and I manage to get to the end of the day. We’ve had an intense life and now we’re grandparents. Now my biggest regret is that I can no longer see my grandson playing football! I follow him a little now, almost nothing! I would have liked to see my grandchildren become adults. My biggest regret is that I have to die before that happens.”

### 3.2. The Second Theme: Personal Dignity as Characterised by the Values of Personal Success, Hope, and Wisdom

The dignity dimension of role preservation intersected with the maintenance of personal pride, which shared the same fundamental values. These included almost all the dimensions of the QCMHV: Personal success (*f* = 11); pride in personal ability (*f* = 10); benevolence (*f* = 9); being able to help others (*f* = 7); self-direction (maturity) and independence (*f* = 7); power (i.e., authority, understood as the right to lead or command; *f* = 4); recognition from others (*f* = 3); and tradition (*f* = 3). One quote from the fourth participant aptly represented these values: “Marriage has made me take an important step in personal growth and maturity! Building a family of one’s own makes you understand what things really worthwhile and what things are merely dazzles. As a father, I think I have given my children an example of what a man must do to be considered reliable. I’m talking about the value of responsibility, wherein one must know how to manage both work and personal relationships.” Success appeared for a sixth person as a sense of personal pride in his own abilities (*f* = 7), which he elaborated as he looked through photos of himself giving the opening speech for the 1980 Moscow Olympics: “Yes, an incredibly moving memory. I made that presentation in front of people much more important than me. I was terrified, I wanted to hide, so much so that I was ashamed. But that’s me, yes, that’s what I did.” Similarly, The fifth person spoke of his political role: “In [terms of] political activity, I have also organized many very successful public events. I have always been the main organizer, and almost every year an important political figure participated, making a significant contribution to the public discussion. It was all extremely exciting because the hope was that everything would go well. But what hard work! And yet, I would do it all again, just everything, because I was the one who guaranteed the success of these important moments of local political life.”

The maintenance of personal pride assumed more specific characteristics, operating through the values of maturity and universalism, which involve a mature understanding of life (*f* = 11). This was exemplified by the third participant: “What I wanted most was to reach any aim together with my wife. We wanted to become grandparents, and this is what happened. We grew up together with our children and we set an example for them, so they, in turn, became parents. All this is really important. In the past, unlike today, marriage was the only way to get out of the house, to live without parental oppression. Parents used to rule all the time, and children had to obey even when their choices were questionable. Marriage meant getting rid of this oppression. My wife and I, on the other hand, were able to leave our children free, and our ability allowed them to choose their family freely, out of love. My wife and I have reached the goals of 25 and 40 years of marriage. These are important stages that have made us understand the true values of life.”

Similarly, the sixth person said, “I realize that suffering makes people grow. If I think back to who and how I was, eh, I can say that I have really changed and learned a lot! The most important thing I have learned is the value of respect and listening to others. Slowly, I discovered that each person could teach me something new, useful or different!”

### 3.3. The Third Theme: Hope and Generativity

The dignity dimension of generativity/legacy was related to hope, and both were associated with benevolence, understood as grasping the meaning of life (*f* = 3), and universalism (*f* = 3). To this effect, the fourth participant said, “With my example, I think I have made [my children] understand what [life] means, and how one should never underestimate the commitment that work requires or the effort necessary to […] keep the family going. I know my kids have recognized my ability in all this.” In the same way, the second person interviewed stated, “When you get to this point, everything you’ve lived through before seems like nothing. You wish you still had time left, days to live, to be with loved ones. But what consoles me is knowing that what I have done and given is kept in my daughter’s memory and heart.” However, it was the third participant, once again, who summed up this sentiment very well, turning, towards the end, to his wife: “Do you see how well the grandchildren are coming along? The children are fine: they have a home, a decent job, a comfortable future! At the end of my life, I can say we’re a good family, and when we can, we try to stay close and be good together. Even the sisters-in-law, between them, are almost two sisters, maybe even more! What could be more exciting than that? This is what counts.”

Generativity was linked to the present self (*f* = 6), as evidenced by the seventh participant’s narrative: “I believe I have left an important mark in other people’s lives! I notice it in some people who make reasons like me whereas before they didn’t […]! I know I have influenced a lot of people because my reasoning has never been superficial. If you reason, you make other people reason, too. This ability to think about certain things will be what I leave to them.” The first person interviewed was convinced that he had taught his children that “[l]oving and respecting your parents is the most important thing, as there are no other people in the world who love you in the same way. A parent will always be there, even in the face of the worst difficulties. However, I always advise my children not to give up easily in life, to know how to face every difficulty, because, by facing them, you learn and move on in life. Every difficulty is a lesson!” The fourth participant’s narrative highlighted the relationship between universalism and wisdom, and he stressed that “[o]ne should not be afraid to face life! This does not mean facing it head-on, exasperatedly, but with a certain philosophy, respecting the fundamental values in which one believes and pursuing with conviction the objectives chosen on the basis of those values. The fatigue is significant, but the satisfactions of life reward every suffering! Without sacrifice, there is no satisfaction! The rest is just illusions!”

### 3.4. The Fourth Theme: Final Opinion on the Experience

The final theme reflected participants’ opinions regarding the DT experience. All participants felt satisfied with the therapy because it allowed them to reflect on the meaning of their life and to rediscover its deep value and beauty. As the second person interviewed explained, “In the last year, I had forgotten the happy, beautiful moments; I had erased them as if they never existed. Now I can remember them, because there were some! Looking at the photos and talking with you allowed me to take out from the trunk of my memory everything important and significant that I did in life. I risked not thinking about it anymore. It will be a nice memory for my daughter. It’s like leaving something behind!” Almost everyone would recommend the intervention to people in the same condition, as it offers the opportunity to reduce suffering and anguish. The fourth participant stated, “This dialogue helped me to open up a little more to my condition, to get rid of certain worries and sadness related to the disease. I forgot almost all the facts of my life and their importance. The cancer deletes memories and feelings because it causes unbearable suffering, and you cannot think of anything other than the cancer. Looking at the album, I could change the direction of my thoughts and remember my whole life.” The third participant stated that, “when you die, it is like you never existed because your memories cease to exist. This document testifies that I existed, I did a lot of things. I believed in such values, such principles! I was someone who did something good!”

Three wives and two nurses who participated in the treatment commented on the whole experience, reporting their personal points of view. For example, the fourth participant’s wife, said, “I was struck by all the memories my husband shared. While he was talking, I saw our whole lives passing in front of me! Many things I didn’t even think about anymore, and he made me understand how they had an important value for him.” The fifth person’s wife, said, “Listening to him allowed me to discover how much value our life together had. I had never thought about it, especially in these recent times when the disease totally absorbed us. I understood that, regardless of mistakes, failures and impotence, our life has been a life that deserves to be told, remembered, listened to. It has been a good life that deserves to be appreciated!” The fourth participant’s wife, shared: “Telling [the story of our] life with the photos in front of us makes us see things in another way. It helps to reconstruct the facts as they really happened, especially by talking about them together. Many times, things are remembered in different ways. Now after going to look for the photos, your whole life passes in front of you and you see things in a different way! Photographs allow you to reflect on what happened. This is something you never do, and, in this way, you lose the life that has passed.”

According to the second person interviewed’s nurse: “This therapy allows her to close the last moment of life with a sense of inner peace, which originates from the awareness of having left something good, a sort of moral legacy! For those who die, it is important to know that someone will think about and learn from them. This intervention makes it possible to solidify the patient’s dignity because they feel listened to, because they feel that their experiences and emotions interest someone. This is exactly what the dying person needs: to feel like a whole person again.”

Finally, Carla—the individual responsible for all the participants’ nursing service—stated that: “What impresses me, what impresses me a lot, is that the terminally ill can see beyond death, and this allows many problems that had previously been left unresolved to be solved. What really excites me is that family members can remember their loved ones in a new way, not just focusing on the final pain. With this therapy, they can overcome the memory of the last phase of illness and suffering, which so often erases all previous life. I really like the idea that [the patients] can give back the life they have already lived and not let it be erased by the illness.”

## 4. Discussion

People approaching the end of their life are willing to talk about a wide range of experiences, beliefs, and relationships when given the opportunity to do so [49]. In our study, all participants reported autobiographical themes of great depth and relevance, most of which were previously observed by Chochinov et al. [6], namely, periods of passage, often marked by rituals (e.g., marriages, bereavement), life lessons learned, expressions of remorse, hope, and dreams, requests for forgiveness, challenges that have been overcome, declarations of love for family and friends, and personal development in the context of family life. The latter theme was prevalent in all the texts because most important relationships were experienced within this context, and it encapsulated the greatest number of values [30,50,51]. This result confirms the importance of expressing one’s values at the end of life. Inviting patients to reflect upon their values seems to facilitate the attribution of meaning to what they have already experienced. Furthermore, it provides a means for patients to fulfil their need to reach out to others and transmit their wisdom, thereby extending its influence over more than one generation [11]. The provision of a final opportunity to say what is valued prompted our participants to reread their entire lives through the lens of achieved wisdom, the ultimate goal that every human being implicitly yearns for.

The values that proved most significant were benevolence, stimulation, universalism, self-direction, safety, personal success, and power. This sequence of values is partly confirmed by the work of Schwartz and Bilsky [35,39] on healthy people and the work of Martin et al. [52] on individuals in palliative care. As with the latter study, our study suggests that people in palliative care attach less importance to power and success and more importance to benevolence, universalism, and self-direction. Therefore, values shift towards self-transcendence (universalism, benevolence) at the end of life, which indicates a connection with qualities that last beyond life in the world [53,54,55].

Finally, we hypothesize that the PDI did not yield significant results because the participants were patients in home care. In reality, their condition did improve following the intervention because fatigue and drowsiness were significantly reduced. Based on the literature, which considers this particular experience at the end of life [56,57,58,59,60], and given the great satisfaction patients, partners, and caretakers expressed through the dialogues, we can interpret this result as stemming from a resumption of interest in life experiences.

A particular observation must be made with respect to the use of photographs from family albums, the majority of which portrayed the participant together with the people closest to them (relatives, friends). This element made it possible for participants to deeply probe and recognize the values that led to those experiences they considered pivotal in their lives [24,25]. From this point of view, DT shares many qualities with PhotoTherapy, as it allows patients to discover and articulate their own inner value system and related beliefs, self-evaluations, personal judgements, and resultant expectations, which will be used to reinterpret past events and measure future changes.

## 5. Conclusions

The time before death can be an opportunity to gain deep insight into one’s own life and the lives of others. In this sense, for the terminally ill, DT proves to be a very important intervention for managing the last phase of life, restoring to them an important horizon of intrinsic, existential values, and the autonomy and efficacy to define the meanings to be attributed to their lives. This study confirms the importance of personal values, acknowledging that the deepest request of the dying is precisely to end life without feeling that the pain and effort of their final days had depleted the meaning of their lives. The study also confirms the importance of using the family photo album to facilitate recollection, enabling patients to reopen windows that time had closed and to look at past experiences in order to grasp a sense of unity—something that values define with precision.

### Limitation and Future Studies

One limitation of the research is the limited number of people involved. In addition, this study’s results do not indicate if dignity dimensions and values overlap in the same ways in other cultural contexts. Furthermore, statistical analysis should reveal whether these correlations varied with age, gender, education, and economic level. Another important limit is that we shadowed the Chochinov research protocol without utilizing any psychological tool to determine the effect. Further studies could improve this observation, including appropriate psychological scales and possibly a control group. Moreover, the research design should involve a larger number of patients, with wider cultural contexts or a larger group of familiars (family members, friends, medical professionals). Finally, future studies should also compare the relative efficacy of photo-mediated DT with other end-of-life interventions.

## Figures and Tables

**Table 1:** Participants’ characteristics.

Variables	Frequency (%)
Gender:	
male	5 (71%)
female	2 (29%)
Age (years) ^	63 (range 44–79)
School attendance (years) ^	11 (range 5–16)
Nationality:	
Italian	5 (71%)
Albanian	1 (14%)
Moldovan	1 (14%)
Civil state:	
married with children	5 (71%)
married without children	1 (14%)
single with children	1 (14%)
Job:	
worker	2 (29%)
retired	5 (71%)
Pathology:	
cancer	6 (86%)
neurodegenerative (SLA)	1 (14%)
Cancer site:	
lung	1 (14%)
liver	2 (29%)
nasopharyngeal	1 (14%)
angiosarcoma	1 (14%)
pancreatic	1 (14%)
Years from diagnosis receipt ^	2 (1–4)
Length of meetings (days for person) ^	6 (range 4–9)

^ Mean and range of values are reported for this variable.

**Table 2:** Descriptive statistics and Wilcoxon’s test to compare pre-test and post-test for Edmonton Symptom Assessment Scale (ESAS) and Patient Dignity Inventory (PDI).

Variable	Pre-Test		Post-Test		Wicoxon’s Test Results			
	M	SD	M	SD	Number of Negative Ranks ^	Number of Positive Ranks ^^	z	*p*-Value
ESAS								
Pain	4.57	2.07	4.14	2.34	1	5	−0.95	0.339
Fatigue	6.29	2.63	5.14	2.12	0	4	−1.84	0.066
Nausea	2.14	2.79	1.29	1.80	2	3	−0.96	0.336
Depression	4.57	1.51	4.00	2.00	1	4	−0.96	0.334
Drowsiness	5.00	2.65	3.86	2.79	0	4	−1.84	0.066
Insomnia	3.29	2.43	2.43	3.10	2	4	−0.85	0.395
Anxiety	5.43	2.57	5.00	2.65	3	3	−0.1	0.914
Shortness of breath	2.86	3.08	2.71	3.59	2	2	−0.18	0.854
Appetite	3.14	2.41	3.57	1.62	3	1	−1.13	0.257
Discomfort	4.57	2.37	4.71	1.25	3	4	−0.08	0.932
Other	7.00	1.79	6.50	1.22	2	4	−0.63	0.524
PDI								
Existential Distress	25.57	4.72	26.57	5.62	3	2	−1.21	0.223
Psychological Distress	18.71	2.98	17.86	4.26	2	3	−0.67	0.500
Physical and Performance Distress	10.86	2.67	11.29	3.86	2	3	−0.13	0.890
PDI Total score	56.86	7.49	57.71	12.05	4	3	−0.33	0.734

^ Number of negative ranks refers to subjects with Pre < Post; ^^ Number of positive ranks refers to subjects with Pre > Post.

**Table 3:** Intersections between Quasi-Circumplex Model of Human Values (QCMHV) and Model of Dignity in the Terminally ill Patient (MDTP).

	Chochinov’s Dignity Dimensions	Continuity of Self	Role Preservation	Pride Preservation	Posthumous Concerns	Hope Optimism	Generativity Legacy Inheritance
Schwartz’s Values							
**Auto direction**	growth/maturity	X	X	X			
	Independence	X		X			
**Stimulation**	exciting life	X	X				
	diverse life	X					
**Benevolence**	stable and mature romantic relationships	X	X	X		X	X
	help others	X	X	X			
**Universalism**	wisdom	X	X	X	X	X	X
**Personal success**	Being able to do	X	X				
	pride in one’s abilities	X	X	X			
**Security**	safety arising from family	X	X				
	family safety	X				X	
**Hedonism**	enjoyment of life	X					
**Tradition**	respect and transmission of values	X	X				
**Power**	power		X				
**Conformism**	respect and transmission of values						
